# Pediatric vocal cord mobility: Translaryngeal ultrasound application for resource-limited laryngologists

**DOI:** 10.4102/jcmsa.v2i1.59

**Published:** 2024-03-15

**Authors:** Divya Ramyead, Fiona Kabagenyi, Sandhia Padayachee, Marc Jordaan, Shazia Peer

**Affiliations:** 1 Division of ENT Surgery, Faculty of Health Sciences, University of Cape Town, Cape Town, South Africa; 2 Division of ENT Surgery, Red Cross War Memorial Children’s Hospital, Cape Town, South Africa; 3 Department of ENT, Faculty of Health Sciences, Makerere University, Kampala, Uganda; 4 Department of Paediatric Intensive Care (PICU), Faculty of Health Sciences, Sydney Children’s Hospital, Randwick, Sydney, Australia; 5 Department of Radiology, Morton and Partners Radiology, Cape Town, South Africa

**Keywords:** translaryngeal ultrasound, flexible fibreoptic laryngoscopy, nonaerosol-generating procedure, noninvasive, vocal cord mobility, paediatric airway

## Abstract

**Background:**

Flexible fibreoptic laryngoscopy (FFL) is currently the gold standard for assessment of true vocal cord (TVC) mobility but is invasive and not without risk. The aim of this study was to determine the accuracy of an application-based translaryngeal ultrasonography (TLUS) as a screening tool for mobility of TVCs and to assess the feasibility of its use by an otolaryngologist not formally trained in ultrasonography.

**Methods:**

Forty children were recruited at the ear, nose and throat (ENT) clinic at Red Cross War Memorial Children’s Hospital (RCWMCH). The first author (DR), an ENT trainee, was trained by a consultant radiologist (MJ) on the use of an ultrasound probe to assess TVC mobility. Two qualified ENT specialists (neither trained in ultrasonography) consented to evaluate TLUS and FFL videos for TVC mobility.

**Results:**

In total, 135 videos were obtained from 40 participants. Ages ranged from 10 days to 9 years, and the genders were equally represented. The overall accuracy of TLUS evaluation was 95.5% (sensitivity of 100%, specificity of 60%). The reliability of TLUS when compared to FFL showed a *p* < 0.001 and a 100% agreement between ENT specialists evaluating the shared videos.

**Conclusion:**

Our study shows TLUS to be a reliable method of assessing TVC mobility.

**Contribution:**

Translaryngeal ultrasonography is portable, noninvasive and easy to use, making it a potentially useful screening tool for practitioners other than radiologists, for example, otolaryngologists, who have a good understanding of laryngeal anatomy, especially in resource-limited settings, where FFL might not be readily available.

## Introduction

Awake flexible fibreoptic laryngoscopy (FFL) is currently the gold standard for assessing true vocal cord (TVC) mobility and upper airway structures. However, FFL is invasive and is an aerosol-generating procedure, not without risk.^1^ The challenges with children include lack of patient cooperation, difficulty obtaining a sustained image, poor glottic view due to larger supraglottic structures and obstruction from secretions.

The coronavirus disease 2019 (COVID-19) pandemic, together with an increase in airborne diseases, has presented otorhinolaryngologists worldwide with unprecedented challenges regarding the safety of aerosol-generating diagnostic procedures. There has subsequently been growing interest in safe and effective alternatives.

Translaryngeal ultrasonography (TLUS) is a potential alternative or adjunct to FFL for assessing mobility of the TVCs and is currently being used as an initial screening tool in many centres internationally. It has been shown to be reliable for assessing vocal fold mobility and structural abnormalities of the airway.^2^ It has also been used in intensive care units (ICUs) and for perioperative assessment in patients undergoing cardiac, thyroid or head and neck surgery – procedures that carry potential risk of injury to the recurrent laryngeal nerve, the nerve responsible for abduction of the TVCs, and that if injured can cause significant airway symptoms.^3^ Translaryngeal ultrasonography is noninvasive, radiation-free and easy to use.

Although frequently performed by radiologists, studies have shown that it is a feasible modality even when performed by clinicians not trained in ultrasonography, yielding similarly accurate results.^4^ In addition, the emergence of increasingly compact and cost-effective ultrasonographic machines, and translatable technology onto portable devices like tablets, has resulted in greater interest in the role of ‘point-of-care clinician-performed TLUS’, and in our context, airway specialists in the outpatient clinic, and in perioperative and ICU settings. This is especially relevant for clinicians working in resource-constrained settings who benefit from such diagnostic tools.

The aim of this study was to evaluate the accuracy of application-based TLUS as a screening tool for TVC mobility in children in a resource-limited setting. Consequently, it determines the feasibility of its use by an ear, nose and throat (ENT) specialist, not trained in ultrasonography, but who was coached by a qualified radiologist in basic technique and interpretation.

## Research methods and study design

### Study design and setting

A prospective cohort study was carried out at Red Cross War Memorial Children’s Hospital (RCWMCH) in two phases from 01 February 2022 to 30 April 2022.

### Study participants, inclusion and exclusion criteria

In Phase A of the study, 40 children attending the ENT surgery outpatient’s department (OPD) at RCWMCH were recruited. Children aged 13 years or younger who attended the ENT OPD for an upper airway assessment and subsequently had a diagnostic FFL for various indications (upper airway obstruction, dysphonia or dysphagia) were included. Children requiring oxygen, with any airway instability, and/or children with behavioural disorders were excluded.

In Phase B of the study, two qualified ENT specialists with more than 2 years of clinical experience evaluated TVC mobility from 10 s looped videos of the two modalities, namely FFL and TLUS.

### Study procedure

#### Phase A

Laryngeal anatomy, technique and key points of reference were practiced with an experienced radiologist, who demonstrated and then assessed DR’s performance. Following this, and prior to commencement of the study, investigator DR performed a minimum of 10 TLUS studies under supervision on children with normal airway anatomy and function. Positioning of the patient, placement of the ultrasound probe, identification of landmarks and assessment of vocal cord mobility were standardised during this informal training.

**FIGURE 1:**
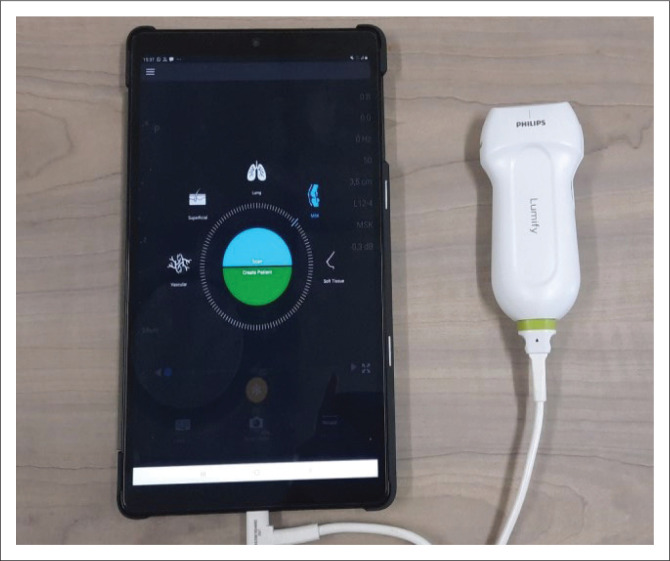
Handheld transducer attached to portable device displaying ultrasound application.

A Lumify^®^ application-based portable ultrasound system (Philips Ultrasound, Inc, Bothell, Washington) was used to perform TLUS. The system comprises of a handheld transducer (Lumify^®^ ultrasound probe, an L12-4 linear array transducer with 12-4 MHz bandwidth) that connects directly to a tablet or mobile phone. The application is free for download from Apple/Android^®^ (Figure 1). A Samsung^®^ Galaxy Tablet S3 (Samsung Group, Ridgefield Park, New Jersey, United States [US]) was used in the study.

### Translaryngeal ultrasonography technique

Participants were placed in supine position; a small shoulder roll was used for optimum laryngeal position. The neck was exposed from chin to manubrium and skin gently wiped clean. Using contact jelly, the linear probe was then placed in the midline over the anterior larynx in a transverse position over the anterior aspect of the thyroid cartilage.

In the case of difficult visibility, a lateral approach was attempted, with the probe placed transversely on the right and then left side of thyroid cartilage.

The mobility of the TVCs was recorded passively during quiet respiration to avoid arytenoid movement caused by more forceful movement, and where possible, actively with the child vocalising the letter ‘e’. The diagnostic criterion for vocal fold paralysis used was asymmetric abduction and adduction movements of the true vocal folds (TVF) during phonation.^5^ Where possible the child was asked to temporarily stop breathing to allow the operator to appreciate adduction of the vocal folds to the midline. True vocal cord paresis was seen as decreased movement of TVF while paralysis was seen as no movement. The vestibular folds (‘false vocal folds’) lie above and slightly lateral to the TVF. They are not involved in phonation. They are composed of echoic fat and are seen as thick, hyperechoic bands in the shape of an inverted letter ‘v’ on ultrasound. On the other hand, TVF are composed of muscle and are much thinner and more hypoechoic on TLUS.^6,7,8^

**FIGURE 2:**
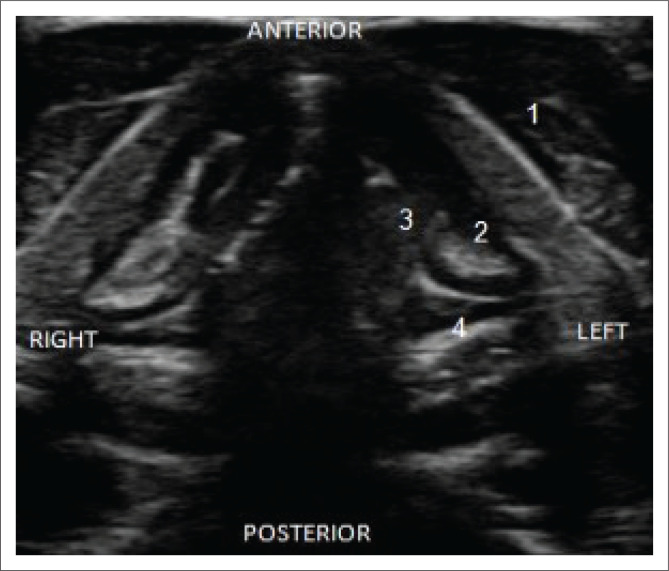
Translaryngeal ultrasonography of vocal cords in abduction where the following anatomical structures are visualised and numbered. (1) Thyroid cartilage, (2) left false vocal cord, (3) left true vocal cord and (4) arytenoid cartilage.

**FIGURE 3:**
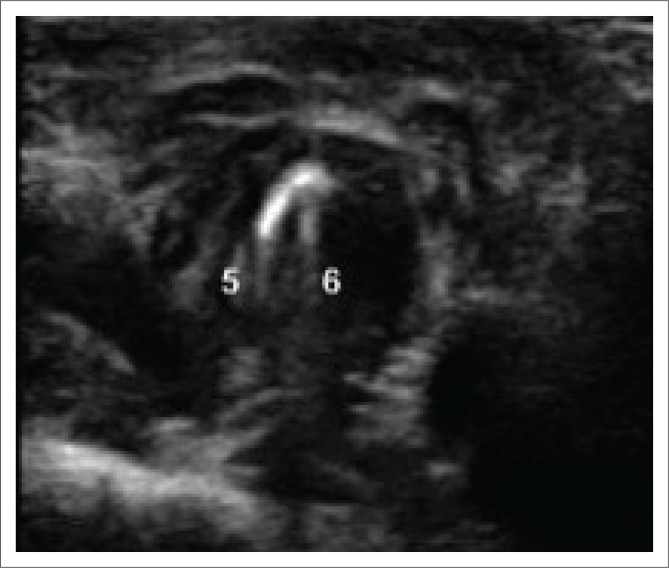
Translaryngeal ultrasonography of left vocal cord palsy where the following anatomical structures are visualised and numbered. (5) Right true vocal cord. (6) Left true vocal cord (fixed in midline).

The most described sonographic landmarks are the arytenoids (ART), TVF and false vocal folds (FVF)^6,9^ as seen in the study images (Figure 2 and Figure 3).

Following consent, participants underwent TLUS, performed by the first author (D.R.).

### Video selection and preparation

Once the TVCs were adequately visualised, a 10 s video recording was made. All videos were of a suitable clinical quality for viewing the anatomy and mobility of the TVCs. Video recordings and related data during each patient TLUS assessment were stored in a password-protected folder on the Samsung^®^ Galaxy Tab S3 (Samsung Group, Ridgefield Park, New Jersey, US).

Each participant had one FFL video (anonymised to P01FFL-P40FFL) paired to a minimum of three TLUS videos (anonymised to P01TLUSa, TLUSb, TLUSc–P40TLUSa, TLUSb, TLUSc). Some participants had up to six TLUS videos. A total of 135 TLUS videos and 40 FFL videos were obtained from the 40 participants. These videos were further anonymised and given a random video study number to prepare for performing Phase B of the study that is evaluation of the two modalities for TVC assessment.

#### Phase B

Consent was obtained from both evaluators prior to commencement of the study. Evaluators also oriented to laryngeal anatomy and the position and mobility of the TVCs on the ultrasound and videos prior to commencement of their participation in the study. The evaluators were asked to imagine a line drawn from the anterior commissure (apex of where both TVCs meet anteriorly), down the centre of the screen to divide the image of the larynx into two halves – left and right. They were then asked to report on the TVC mobility of each side. The evaluators were given 60 s per video to answer the following questions:

- Were both TVCs mobile?
- Was a unilateral TVC immobile?
- If so, which side was immobile (right or left)?
- Was there a glottic gap in cases of palsy?
- Evaluators were also asked to comment on other findings.

### Study analysis

Patient demographics included age, gender and any underlying condition, which were presented descriptively. Categorical variables were presented as numbers, proportions and percentages. The proportion of ‘failed assessments’, where the evaluator is unable to clearly visualise the anatomy in each video, was calculated. The proportion of anatomical landmarks identified correctly on TLUS when compared to FFL was calculated. The proportion of cases in which abnormal mobility (unilateral TVC paresis) was present and correctly identified was also calculated. Fisher’s exact test was used to test statistical significance.

### Ethical considerations

Ethical approval was granted by the Human Research Ethics Committee of the University of Cape Town (HREC 202/2021). Written consent was obtained from parents. A redcap database was used to maintain the confidentiality of patients.

## Results

A total of 40 participants aged 10 days to 9 years (median, interquartile range [IQR] = 3 years) were included in the study, and genders were equally represented (Table 1). Normal mobility of the TVCs was noted on FFL in 37/40 (92.5%), with the remaining 3 (7.5%) having a unilateral TVC palsy. The overall accuracy, sensitivity and specificity of TLUS evaluation were 93.3%, 100% and 60%, respectively (Table 2). Although the proportion of cases in which normal mobility was correctly identified was 93.3% (*n* = 120/135), the proportion of cases in which abnormal mobility (unilateral TVC mobility) was present and correctly identified was 100% (*n* = 135/135). It was noted that the evaluators detected the correct mobility by 100% on FFL. On the other hand, from TLUS videos, 40% (*n* = 6/15) of cases with immobile vocal cords were perceived as mobile, while 100% of cases with mobile cords (120/120) were correctly identified. The rule of thumb says that for a test to be useful; the sensitivity and specificity should be more than 1.5. Our study had a value of 1.6, proving TLUS to be useful. The reliability of TLUS when compared to FFL showed a *p* < 0.001 and there was 100% agreement between ENT specialists evaluating the shared videos.

**TABLE 1:** Demographic profile of participants’ age, gender and underlying condition.

Demographic profile	Total participants (*n* = 40)			
	Median	IQR	*n*	%
**Age (months)**	41.1	13.6–74.3	-	-
Range	-	1.70–210	-	-
**Gender**				
Female	-	-	20	50.0
Male	-	-	20	50.0
**Underlying condition**				
Upper airway	-	-	-	-
Laryngeal	-	-	-	-
Laryngeal cleft	-	-	1	2.5
Laryngeal papilloma	-	-	2	5.0
Laryngomalacia	-	-	1	2.5
Croup	-	-	1	2.5
Laryngopharyngeal reflux	-	-	1	2.5
Nasal	-	-	-	-
Adenoid hypertrophy	-	-	22	55.0
Allergic rhinitis	-	-	1	2.5
Rhinitis and adenoid hypertrophy	-	-	1	2.5
Sleep apnoea	-	-	1	2.5
Central	-	-	-	-
Cerebral palsy	-	-	1	2.5
Traumatic brain injury	-	-	1	2.5
Myelomeningocele	-	-	1	2.5
Subdural hygroma	-	-	1	2.5
Post-cardiac operation	-	-	5	12.5

**TABLE 2:** Results of evaluators’ translaryngeal ultrasonography vocal fold assessment when compared to flexible fibreoptic laryngoscopy vocal cord assessment.

Evaluator	TLUS	FFL true vocal cord assessment						
		Total (*n* = 135)	Mobile (*n* = 120)	Immobile (*n* = 15)	*p*-value Fishers exact	Sensitivity (%)	Specificity (%)	Accuracy (%)
1	Mobile	126	120	6	< 0.001	100.0	60.0	93.3
	Immobile	9	0	9				
2	Mobile	126	120	6	< 0.001	100.0	60.0	93.3
	Immobile	9	0	9				

FFL, flexible fibreoptic laryngoscopy; TLUS, translaryngeal ultrasonography.

## Discussion

There has been a growing interest in neck ultrasonography to visualise TVC movement in recent years. In 1987, Raghavendra et al.^7^ reported that in their cohort of 41 healthy volunteers, TLUS was limited by acoustic shadowing caused by calcification of the laryngeal cartilages. Hu et al.^10^ looked at 229 volunteers with no pathology and found that ultrasonography could ‘quantitatively measure both the true and false vocal cords with good reliability and reproducibility’. A thorough anatomical study of the upper airway was performed by Singh et al.^11^ and showed the ability for TLUS to investigate anatomical variations in the larynx, speech and swallowing abnormalities, to confirm endotracheal tube placement, as a treatment adjunct in percutaneous tracheostomy and cricothyrotomy, and to predict post-extubation stridor and difficult intubations.

Friedman compared TLUS to FFL in correctly diagnosing vocal cord immobility in infants and children.^12^ There are many studies in literature that have since confirmed TLUS as a successful alternative to FFL to evaluate vocal fold mobility in children.^7,8,13,14,15^

In our study, the first author underwent ultrasound training with a qualified radiologist and completed 10 suitable ultrasound examinations before starting with study participants. This training was aimed specifically at screening for TVC mobility and, for the purposes of the aim, is limited as such. Wong et al. evaluated the learning curve of TLUS training in a group of inexperienced ultrasound assessors and found them to be competent in TLUS after the seventh examination, with little to no change in outcome thereafter.^4^

In our study, the accuracy was 93.3%, similar to accuracies of > 90, and 99% – 100% reported in studies by Klinge et al. and Jadcherla et al., respectively.^9,15^ Wang et al. reported an inter-rater agreement of 96% when comparing TLUS to FFL in children and subsequently found TLUS to be a useful diagnostic tool in vocal fold paralysis.^1^ We found an inter-rater agreement of 100% between assessors in our study.

Interestingly, during the TLUS examination, we identified vocal fold movements resulting from the Bernoulli’s effect of air through the glottis during respiration. This is not seen with FFL.

Our study, like similar reported studies, has shown feasibility and utility of TLUS to assess TVC mobility by ENT specialist surgeons in a clinic setting. Translaryngeal ultrasonography is noninvasive and is well tolerated by children and their families.^5^ More specifically, TLUS can be included in practice protocols especially in resource-constrained settings with poor access to expensive FFL. In addition, the ultrasound device is robust and can be easily transported to outreach facilities far from tertiary hospitals, thereby preventing delays in diagnosing airway conditions in children.

### Strengths and limitations

Translaryngeal ultrasonography helps to combat the challenges faced when assessing patients with communicable diseases such as tuberculosis and COVID-19 where aerosol-generating procedures like FFL are considered high risk.^7,16^ Furthermore, TLUS proves to be particularly useful in medically fragile patients or patients with difficult FFL as it does not alter the physiological parameters associated with invasive procedures.^17,18^

While it has been shown to be effective in identifying gross true vocal fold mobility, our study did not investigate the laryngeal anatomy or pathology beyond assessing vocal cord mobility. There are several limitations to the use of TLUS. One of the major drawbacks of TLUS is that it is operator-dependent, and assessments such as the objective movement of the TVF may be subject to interuser interpretation. In the case of ‘clinician-performed ultrasound’, no formal ultrasound training is obtained and there is a learning curve involved for each clinician.^4,16^ However, computer-aided diagnostic applications hold promise for the future by making it possible for individuals with basic ultrasound training and a good understanding of the laryngeal anatomy to assess mobility of the TVCs. Moreover, some studies have noted difficulty diagnosing mobility of the TVCs in children under 12 months of age due to the smaller window of access for the probe and lack of cooperation of younger patients.^19^ Lastly, the grading of the severity of vocal cord paralysis has not been fully reported with TLUS.^3^ Our study was limited by the fact that our cohort comprised 40 participants and only a small proportion had vocal cord paresis.

## Conclusion

Translaryngeal ultrasonography is a noninvasive and useful alternative screening tool to assess TVC mobility. Our study similarly shows promise. While being an adjunctive diagnostic tool, TLUS can potentially reduce the need for but not replace FFL in patients requiring a TVC mobility and an upper airway assessment. It is portability and easy to use. This makes it feasible for otolaryngologists, with no ultrasound training, but who have acquired some key diagnostic and technical skills from a qualified radiologist, to assess vocal cord function. This is the first study to use TLUS as a screening tool in vocal cord mobility assessment in sub-Saharan Africa, a resource-constrained setting, where FFL is not readily available. However, more studies are needed to fully define the place of laryngeal ultrasound in the diagnosis and management of children with airway abnormalities.
